# Comparison of the P300 between maintenance and manipulation conditions in the auditory N-back paradigm among early and late adolescents

**DOI:** 10.1590/2317-1782/e20240368en

**Published:** 2026-07-27

**Authors:** Rajesh Ranjan, Jayashree Sunil Bhat, Mohan Kumar Kalaiah

**Affiliations:** 1 Department of Audiology and Speech Language Pathology, Kasturba Medical College Mangalore, Manipal Academy of Higher Education, Manipal, India.; 2 Nitte Institute of Speech and Hearing, Nitte (Deemed to be University) - Mangalore, India.

**Keywords:** Working Memory, Cognitive Functions, Cognitive Tests, P300, Event-related Potentials, Adolescents

## Abstract

**Purpose:**

The P300 recorded using N-back tasks could be used to identify brain regions involved in the maintenance and manipulation demands of working memory. The difference in brain activity between zero-back and one-back conditions highlights areas activated during the maintenance phase of the working memory. Similarly, the difference between one-back and two-back conditions reveals brain regions engaged in the manipulation process of working memory. The difference between zero-back and two-back conditions reflects areas activated during both the maintenance and manipulation processes. This study aims to compare the P300 between early and late adolescents in elicited using various N back conditions.

**Methods:**

A total of 48 individuals participated in the study in two groups. Group 1 included 24 late adolescents and group 2 included 24 early adolescents. All participants had hearing sensitivity within normal limits in both ears. The P300 was elicited by presenting stimuli using N-back paradigm in zero-back, one-back, and two-back conditions.

**Results:**

The latency of P300 was shorter and amplitude was larger in late adolescents compared to early adolescents. Further, the amplitude of P300 in maintenance process was highest compared to the manipulation process at all the electrode sites. However, this difference was not statistically significant between groups across conditions.

**Conclusion:**

The present study shows that the P300 amplitude is not significantly different between early and late adolescents across the N-back conditions.

## INTRODUCTION

The P300 is a type of event-related potential (ERP), characterised by a large positive peak at a latency of 300 msec after the stimulus onset^(1)^. It can be recorded using a variety of acoustic stimuli, and the P300 elicited using acoustic stimuli is referred to as auditory P300. A wide variety of acoustic stimuli can be used for eliciting the auditory P300, which includes speech stimuli such as vowels^(2)^, syllables^(3)^, and words^(4)^, and non-speech stimuli such as click^(5)^ and tones^(6)^. The P300 is commonly used as a marker for cognitive functions such as attention, decision making, and memory^(7)^. Superior cognitive functions are linked to shorter latencies and larger amplitudes of the P300^(8-12)^. Conversely, smaller P300 amplitudes are linked to poorer performance on various cognitive tests with different information processing demands^(9)^. Typically, the P300 is elicited by presenting stimuli in an odd-ball paradigm, however, it can also be elicited by presenting stimuli in the N-back paradigm.

The N-back paradigm is commonly used in research settings to assess working memory. Working memory is a type of cognitive function responsible for storing information temporarily and processing of stored information^(13)^. It plays an important role in the daily lives of individuals for cognitive tasks such as planning, problem-solving, reasoning, learning new skills, and academic performance^(14-22)^. Working memory involves two distinct processes, one for maintaining temporary information in a readily accessible state, and another for manipulating the stored information to guide future behavior. Specifically, maintenance encompasses the storage, monitoring, and comparison of information within working memory, while manipulation involves reorganizing and updating the information in memory sets^(23,24)^. Several neuroimaging studies have shown that several brain areas are activated during maintenance and manipulation processes of working memory^(25-28)^. Veltman et al.^(23)^ reported that very similar areas of the brain are activated during maintenance and manipulation processes. However, Smith et al.^(28)^ have found a difference in the activation pattern in the dorsolateral prefrontal cortex of the brain during maintenance and manipulation processes of working memory. Dixon and De Frias and Lindenberger et al. establish that older adults show greater variability in working memory compared to younger adults^(29,30)^. This variation is likely due to the influence of broader cognitive domains such as sustained and executive attention, as well as short- and long-term memory, on the working memory efficiency.

The N-back tasks are commonly used to identify the brain areas activated during the maintenance and manipulation process of working memory^(31-33)^. This approach involves recording brain activation during zero-back, one-back, and two-back conditions. The difference in brain activity between zero-back and one-back conditions reflects the brain areas activated during the maintenance process of working memory. Similarly, the difference between one-back and two-back conditions reflects brain areas activated during the manipulation process of working memory. The difference between zero-back and two-back conditions shows the brain areas activated during both maintenance and manipulation processes. A similar approach could be used to understand the effect of maintenance and manipulation processes on the P300.

Studies investigating the maturational changes of P300 have reported an increase in the amplitude of P300 during childhood, and the amplitude reduces with advancing age in adults^(34,35)^. The increase in amplitude of the P300 in children is associated with the rapid development of cognitive functions such as memory and attention. Further, the reduction in amplitude of the P300 in adults is associated with age-related cognitive changes. The information processing skills, which develop rapidly, may continue to develop during adolescence. Conversely, smaller P300 amplitudes are linked to poorer performance on various cognitive tests with different information processing demands^(9)^. The skull thickness also may be associated with an opposing effect on amplitudes, as smaller amplitudes are associated with a thicker skull^(36)^. Smaller P300 amplitudes are also observed when engaging in complex and elaborate information processing strategies and dealing with complex problems^(37,38)^. Thus, these P300 amplitudes may reflect age-linked cognitive change. During adolescence, the opportunity for extensive learning occurs through educational experiences and schooling. Physical changes in the central and peripheral nervous systems, as well as the motor system, are associated with advancing age^(39)^. Therefore, research was planned to identify age-related changes in P300 latency and P300 amplitude during a maintenance and manipulation condition in N-back task using auditory verbal stimuli among adolescents.

Most P300 studies in literature used tone stimuli and visual based N-back task using speech syllable. There is limited research on P300 using speech syllable as purely auditory N-back task. Research on P300 latency reported in the literature for tone burst (314 ms), and for pure tone, it ranged from 250 to 340 ms^(40,41)^ and P300 latencies for speech stimuli were consistently above 500 ms^(42,43)^. Research had indicated that P300 evoked by speech syllable is not same as it elicited by pure tone in auditory N-back conditions. Studying the P300 difference in brain activity between zero-back and one-back conditions reflects the brain areas activated during the maintenance process of working memory. Similarly, the difference between one-back and two-back conditions reflects brain areas activated during the manipulation process of memory. Understanding the physiological mechanisms behind working memory’s maintenance and manipulation of auditory information, including syllable processing at higher cortical levels, age-specific neural responses will help to design age-appropriate memory training or executive function programs in educational or clinical settings.

## METHOD

### Participants

A total of 48 individuals participated in the study. The group 1 included 24 late adolescents (21 female and 3 male) aged between 18.1 to 24 years (mean = 21.07, SD = 1.55). The group 2 included 24 early adolescents (14 females and 10 males) aged between 12 to 18 years (mean = 15.3, SD = 2.18). The age classification used in the present study was in accordance with Newman and Newman^(44)^, the classification strongly emphasizes on Erik Erikson’s psychosocial theory. All the participants had hearing sensitivity within normal limits (<25 dB HL) at octave frequencies from 250 Hz to 8000 Hz. A 226 Hz probe tone is used to record tympanogram. All participants had ‘A’ type tympanogram with acoustic reflex present in both ears. The tympanometry peak pressure was between -50 daPa and +50 daPa and static compliance was between 0.3 to 1.6 mmho in all the participants^(45)^. None of the participants had history of otologic, neurologic, or cognitive deficits. Further, none of the participants were exposed to hazardous noise and ototoxic medications. To rule out cognitive impairment, the Mini-Mental State Examination^(46)^ was performed on late adolescents and Modified Mini Mental Scale for Cognitive function in children^(47)^ for early adolescents. In group 1 all participants obtained score greater than 29^(46)^ and participants in group 2 obtained score greater than 35^(47)^. A score greater than 24 and 28 indicate no cognitive deficit for group 1 and group 2 respectively. Handedness of participants was assessed using Edinburgh Inventory^(48)^, and all participants were right-handed. This study was reviewed and approved by the institutional ethics board. A written informed consent was obtained from participants prior to their inclusion in the study.

### Recording of the P300

The stimuli for eliciting the P300 were presented using the N-back paradigm in zero-back, one-back, and two-back conditions. Each condition had a total of 300 stimuli, of which 60 stimuli were targets and the remaining 240 were standard stimuli (20% and 80% respectively). Stimuli used for the N-back task included consonant-vowel syllables spoken by a female speaker. A total of eight syllables (/ka/, /ga/, /ta/, /da/, /pa/, /ba/, /na/, and /ma/) were used as stimuli in the study. The syllables were obtained from the corpus used in an earlier investigation^(43)^. In the zero-back condition, the syllable /pa/ served as the target stimulus, and other syllables served as standard stimuli. The participants were instructed to press a button on the response pad using their right index finger as soon as the target syllable /pa/ was heard. In the one-back condition, participants were instructed to press the response button whenever the currently presented syllable was the same as the preceding syllable. In the two-back condition, participants were instructed to press the response button whenever the currently presented syllable was the same as the two stimuli back. In all N-back conditions, the inter-stimulus interval between syllables was fixed at 1700 msec.

The P300 was recorded using the Geodesic EEG System with Net Amps 400 EEG Amplifier (Electrical Geodesics Inc, USA). Participants were instructed to sit comfortably on the reclining chair and reduce head and body movements during the recording. A 64-channel hydroCel geodesic sensor net GSN 64 1.0 was fitted on the scalp. The electrode impedance at each electrode was maintained below 50 kOhms. During the N-back tasks, the ongoing EEG was band-pass filtered (0.01 Hz to 100 Hz) with a 112 ms digital anti-aliasing filter, digitized at a sampling rate of 250 Hz, and saved for offline analysis. The sequence of N-back conditions was randomized. All the recordings were completed in a single session, and a rest period of 5 minutes was provided between N-back conditions as required by participants. The stimuli were delivered to the right ear of participants at 80 dB SPL using ear-tone ER-3C insert earphones.

The offline analysis was carried out using the NetStation toolbox (version 5.2). Initially, the continuous EEG was band-pass filtered between 0.1 Hz and 30 Hz using FIR filter by maintaining the filter slope at 2 Hz. After filtering, bad channel replacement was carried out with default settings. Following this, data were segmented into epochs with a pre-stimulus duration of 100 ms and a post-stimulus duration of 3000 ms. After segmentation, sweeps with large amplitude variations (artifacts) were removed based on the default predefined criteria, for bad channels and eye blinks the (maximum - minimum) amplitude if it was > 100 µV, within window size 640 ms for entire segment, performed a moving average of 80 ms. For eye movements (Maximum - minimum) amplitude was > 65 µV, window size 640 ms, perform a moving average of 80 ms. The channel was marked as bad for all segments if bad channel was greater than 20 percent of segments and marked segment bad if it contains more than 10 bad channel on eye blink and eye movement). Segments with more than 10 bad channels were discarded. Baseline correction was carried out by considering the pre-stimulus activity of 100 ms before the onset of stimuli. Finally, averaging was performed to obtain averaged waveforms for target and standard stimuli. Prior to averaging, the data were referenced to an average reference.

Accuracy of response and reaction time were also measured in the zero, one, and two-back conditions. The accuracy score refers to the percentage of correct identification of target syllables. Reaction time is the duration between the onset of a target syllable and the participant’s button press following its correct identification. These behavioral measures were recorded alongside EEG data during P300 recording. Reaction time provides insight into the speed of stimulus processing and is analyzed in relation to neural markers such as P300 latency and amplitude.

### Data analysis

The amplitude and latency of P300 are highly dependent on the stimulus parameters used to record the P300. Hence, the participant-specific averaged waveforms were grand-averaged together for each electrode site, N-back condition for standard and target stimuli separately, to identify various ERP components. Based on the grand-averaged waveforms, a positive peak between 400 and 1200 msec was identified as the P300 component, based on which the P300 was marked for participant-specific averaged waveforms. The identification of P300 peaks was carried out by two independent audiologists. The peak was considered present only when there was agreement between the two audiologists. The P300 latency and amplitude were measured at electrode locations Pz, Cz, and Fz for each N-back condition. The accuracy score and average reaction time were also noted for zero, one, and two-back conditions.

To investigate the effect of the level of N-back conditions on the P300 (i.e., amplitude or latency), a mixed model ANOVA (parametric) or Friedman (non-parametric) test was used. The choice of parametric or non-parametric tests was based on result of the normality tests (i.e., Shapiro-Wilk tests). When data was normally distributed, a mixed model ANOVA was carried out with N-back condition as repeated measures and group as between-subject factor. On the other hand, a Friedman test was carried out when data was not normally distributed.

Further, the P300 was compared between maintenance, manipulation, and combined maintenance and manipulation demand conditions. The difference in P300 amplitudes between 1-back and 0-back conditions was computed (i.e., one back P300 amplitude was subtracted from the zero back P300 amplitude), which served as the maintenance condition. For manipulation condition the difference of P300 amplitude between two-back and one-back was computed. For maintenance and manipulation, the “2-back vs 0-back” contrast was created, that is P300 amplitude at two back was subtracted by P300 amplitude at zero back.

## RESULTS

**Figure 1:**
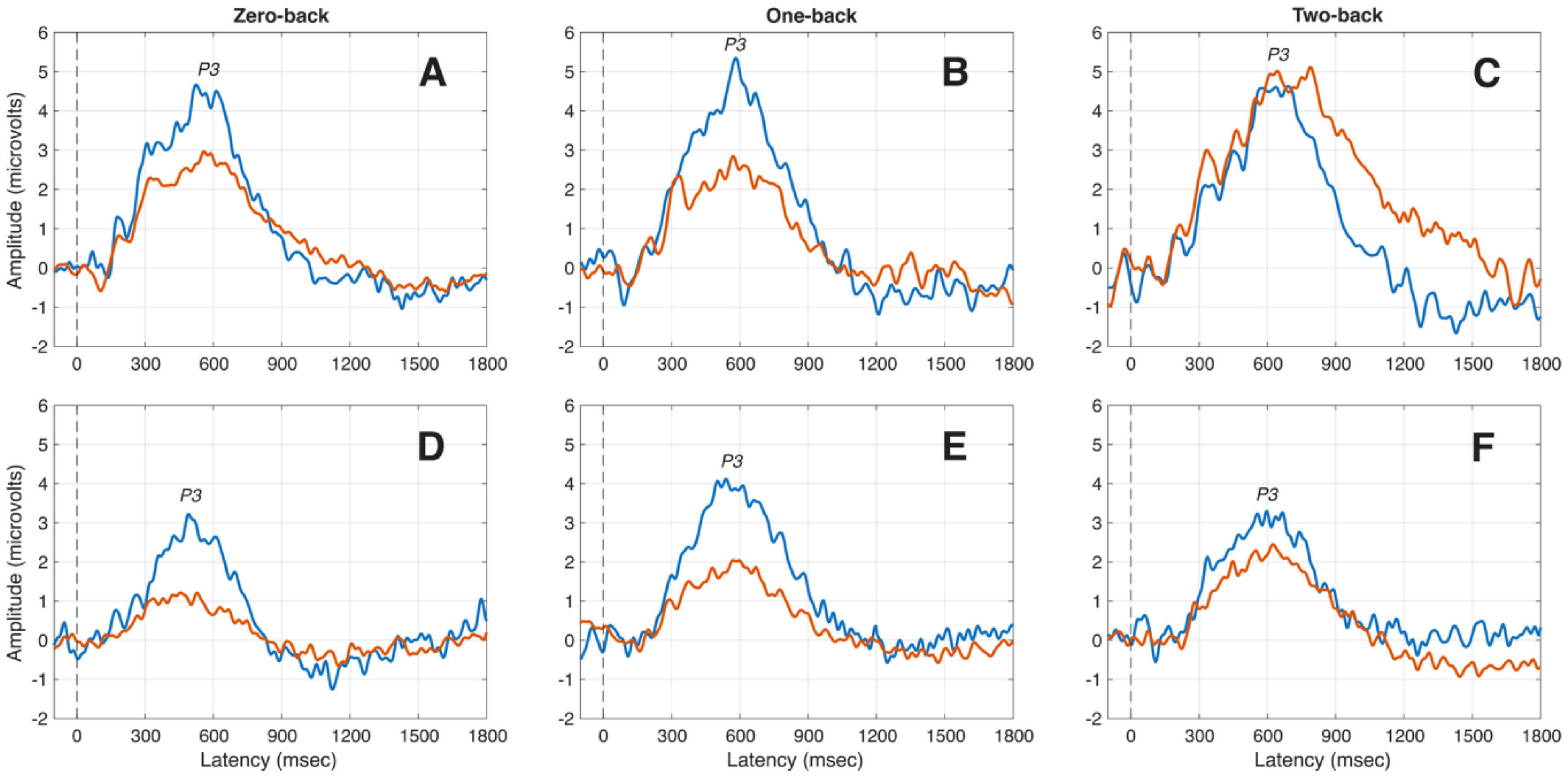
Represents grand-averaged P300 waveforms recorded at the Pz electrode for the target (blue color) and the standard stimuli (red color). Panels A, B, and C show waveforms of zero-back, one-back, and two-back conditions, respectively, for the early adolescent group. Similarly, panels D, E, and F show waveforms in zero-back, one-back, and two-back conditions, respectively, for the late adolescent group.

**Figure 2:**
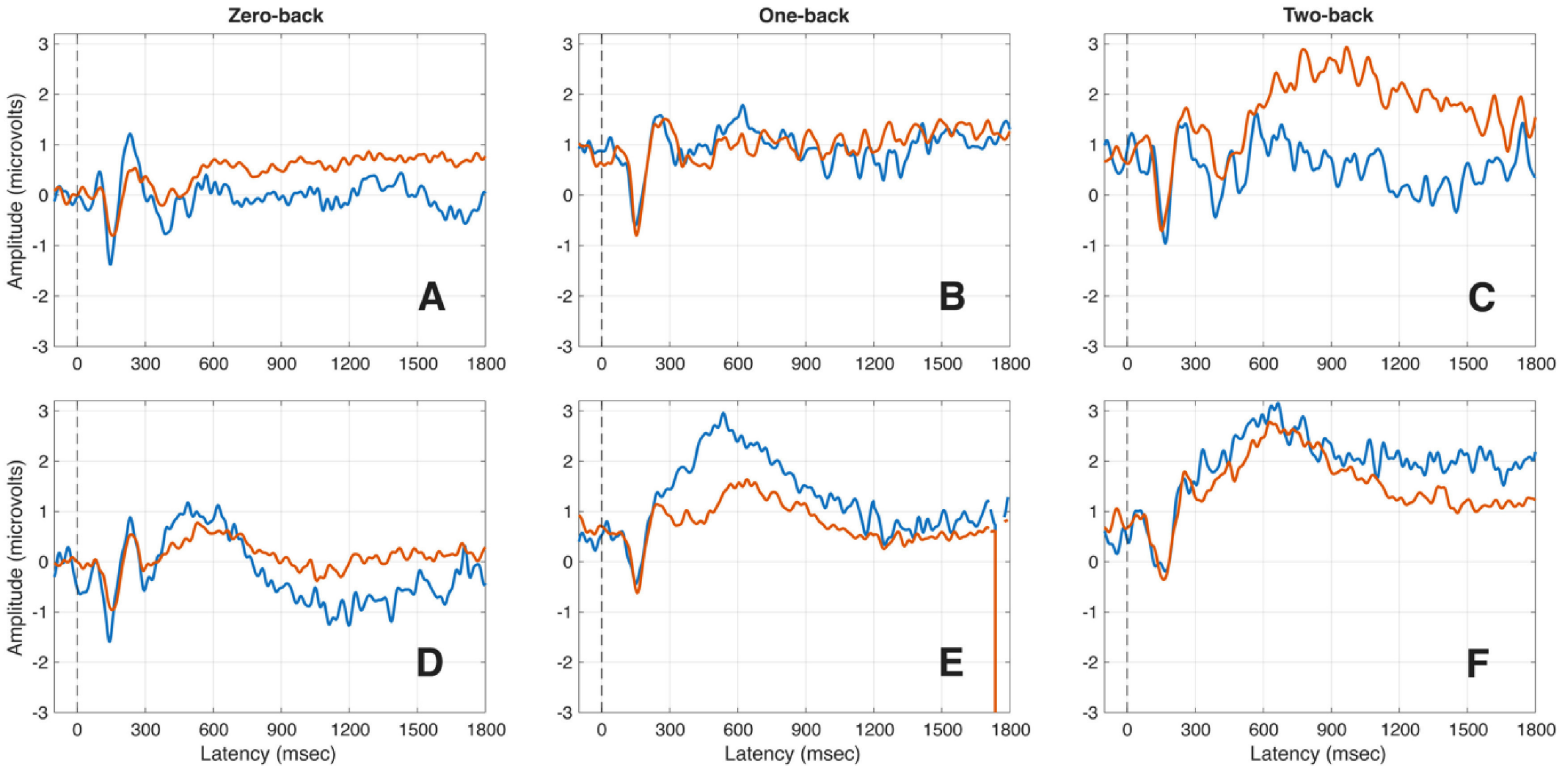
Represents grand-averaged P300 waveforms recorded at the Cz electrode for the target (blue color) and the standard stimuli (red color). Panels A, B, and C show waveforms of zero-back, one-back, and two-back conditions, respectively, for the early adolescent group. Similarly, panels D, E, and F show waveforms in zero-back, one-back, and two-back conditions, respectively, for the late adolescent group.

**Figure 3:**
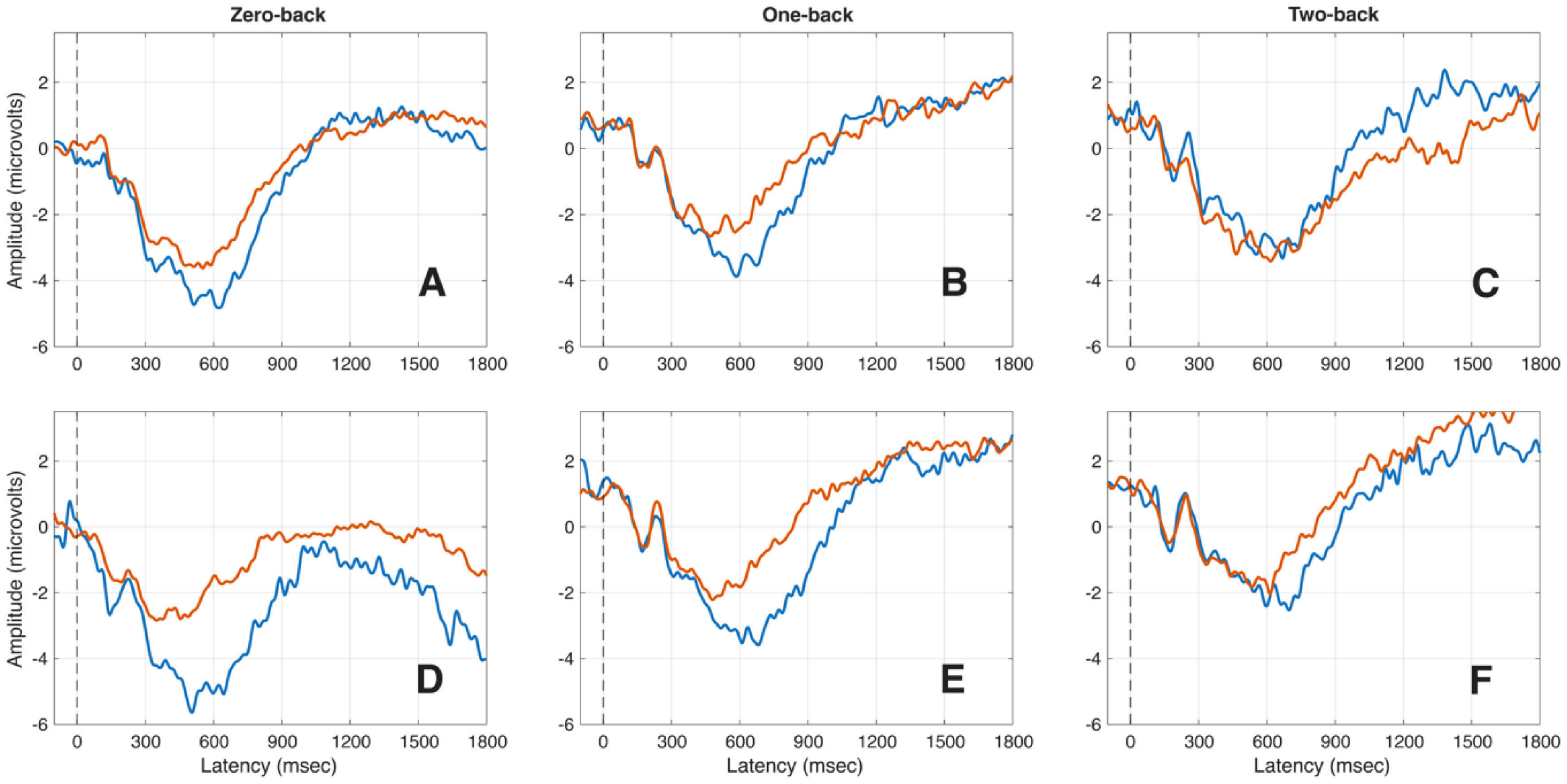
Represents grand-averaged P300 waveforms recorded at the Fz electrode for the target (blue color) and the standard stimuli (red color). Panels A, B, and C show waveforms of zero-back, one-back, and two-back conditions, respectively, for the early adolescent group. Similarly, panels D, E, and F show waveforms in zero-back, one-back, and two-back conditions, respectively, for the late adolescent group.

Results of the N-back task showed that late adolescents outperformed early adolescents across conditions. Accuracy score for correct identification of target stimuli was highest in the zero-back condition and decreased with increasing task difficulty in both groups. The mean accuracy scores were 91% and 98% for early and late adolescents in zero-back condition, 85% and 93% in one-back condition, and 68% and 77% in two-back condition. Similarly, the reaction time was shorter in late adolescents compared to early adolescents across all N-back conditions. The mean reaction times were 798 msec, 804 msec, and 966 msec for early adolescents and 721 msec, 816 msec, and 1057 msec for late adolescents at zero-back, one-back, and two-back conditions respectively. Thus, both groups showed highest accuracy and shortest reaction time in the zero-back condition, followed by the one-back and the two-back conditions. Figure 1, Figure 2, and Figure 3 show the grand averaged P300 waveforms recorded at the Pz, Cz, and Fz electrode sites, respectively, for target (blue color) and standard (red color) stimuli across zero-back, one-back, and two-back conditions.

### P300 amplitude

Table 1 shows the mean amplitude and standard deviation for the P300 across electrode sites and N-back conditions for both groups. The amplitude of P300 was highest in the one-back condition and lowest in the two-back condition in both groups. Further, across electrodes, the amplitude was highest at the electrode Pz, followed by Cz and Fz. The results of the Shapiro-Wilk test showed that the P300 amplitude at the Pz and Fz electrode sites was not normally distributed; on the other hand, the P300 amplitude at Cz was normally distributed in both groups.

**Table 1:** Mean P300 amplitude (in µV) and standard deviation (in parenthesis) for late and early adolescent groups across the electrode sites and n-back conditions

Electrode	Zero-back		One-back		Two-back	
	Late adolescents	Early adolescents	Late adolescents	Early adolescents	Late adolescents	Early adolescents
Pz	4.36 (2.06)	6.15 (4.54)	5.12 (2.35)	6.55 (3.72)	3.90 (1.75)	5.21 (3.73)
Cz	3.58 (1.50)	2.62 (1.34)	4.22 (1.85)	3.41 (1.86)	3.09 (1.29)	2.26 (1.77)
Fz	-6.53 (3.47)	-5.46 (3.06)	-4.7 (3.11)	-4.95 (3.81)	-2.88 (3.37)	-4.53 (2.79)

Note: Amplitude in micro volt (µV).

Thus, the Friedman test was carried out to investigate the effect of the N-back condition on the amplitude of P300 at Pz and Fz electrode sites. Results showed a significant main effect of N-back conditions on P300 amplitudes at Fz [χ^2^(2) = 8.58, *p* = 0.014, Kendall’s W = 0.17] and no significant effect at Pz [χ^2^(2) = 3.2, *p* = 0.19, Kendall’s W = 0.06] in late adolescents. The post-hoc analysis indicated that the P300 amplitude in the zero-back condition was significantly lower (i.e., more prominent) compared to the two-back condition at the Fz electrode site (*p* = 0.004). However, no significant difference was found between zero-back and one-back conditions (*p* = 0.06) and between one-back and two-back conditions (*p* = 0.31). On the other hand, in the early adolescent group, the N-back condition had no significant effect on P300 amplitude at both Fz [χ^2^(2) = 2.33, *p* = 0.31, Kendall’s W = 0.04] and Pz [χ^2^(2) = 3.5, *p* = 0.16, Kendall’s W = 0.07] electrode sites.

Since the P300 amplitude at the Cz electrode site was normally distributed, a repeated measures ANOVA was carried out with N-back conditions as the within-subject factor and group as the between-subject factor. The results indicated no significant interaction between group and P300 amplitude [*F*(2,80) = 0.730,*p*= 0.793, η^2^ = 0.006]; however, the N-back condition had a significant effect on the P300 amplitude at Cz (*F*(2,80) = 8.92, *p* < 0.001, η^2^ = 0.182). The post-hoc analysis indicated that the P300 amplitude at one-back was significantly higher compared to the zero-back condition (*p* = 0.03), and the P300 amplitude was significantly higher in one-back compared to two-back conditions (*p* < 0.001). However, no significant difference was found between zero-back and two-back conditions (*p* = 0.749).

In addition, to compare the P300 amplitude between groups at each electrode site, the Wilcoxon signed test was carried out separately for three N-back conditions. The results showed no significant difference between the groups across N-back conditions at Pz [zero-back (U = 338.5, *p* = 0.29), one-back (U = 348, *p* = 0.21), two-back (U = 329, *p* = 0.39], and Fz: zero-back (U = 329.5, *p* = 0.39), one-back (U = 283.5, *p* = 0.92), two-back (U = 222, *p* = 0.17)]. At the Cz electrode, the P300 amplitude in the two-back condition was significantly higher for late adolescents compared to early adolescents (U = 156, *p* = 0.02); but no significant difference was observed for zero-back (U = 159.5, *p* = 0.05) and one-back conditions (U = 197, *p* = 0.14).

### P300 latency

Table 2 shows the mean latency and standard deviation for the P300 across electrode sites and N-back conditions. The latency of P300 was lowest in zero-back conditions and highest in two-back conditions in both the groups. Further, across electrodes, the latency was lowest at the electrode Pz, followed by Cz and Fz. Prior to further statistical analysis, the latency data of P300 were subjected to Shapiro–Wilk test. The results showed that the P300 latency at the electrode sites Pz, Cz, and Fz were not normally distributed in both groups, except Fz in the late adolescent and Cz in the early adolescent groups.

**Table 2:** Mean P300 latency (in ms). and standard deviation (in parenthesis) for late and early adolescent groups across the electrode sites and n-back conditions

Electrode	Zero-back		One-back		Two-back	
	Late adolescents	Early adolescents	Late adolescents	Early adolescents	Late adolescents	Early adolescents
Pz	599 (112)	585 (93)	640 (98)	634 (108)	660 (115)	661 (83)
Cz	612 (126)	605 (83)	647 (117)	628 (75)	678 (96)	640 (84)
Fz	636 (96)	636 (100)	663 (105)	634 (95)	661 (86)	660 (78)

Note: Latency in millisecond (ms).

Thus, the Friedman test was carried out to investigate the effect of the N-back condition on the latency of P300 at electrode sites, Pz and Cz in the late adolescent group and Pz and Fz in the early adolescent group. For the late adolescent group, the results showed a significant main effect of N-back conditions on the latency of P300 at electrode sites Pz [χ^2^(2) = 10.58, *p* = 0.005, Kendall’s W = 0.22] and Cz [χ^2^(2) = 11.93, *p* = 0.003, Kendall’s W = 0.25]. The following post-hoc analysis indicated that the P300 latency at Pz was significantly higher in the one-back compared to the zero-back conditions (*p* = 0.018), and the P300 latency at two-back was significantly higher compared to the zero-back condition (*p* = 0.012). But no significant difference was found between one-back and two-back conditions (*p* = 1). On the other hand, P300 latency at Cz was significantly higher in the one-back compared to the zero-back condition (*p* = 0.035), and the P300 latency at two-back was significantly higher compared to the zero-back condition (*p* = 0.001). But no significant difference was found between one-back and two-back conditions (p = 0.937).

In contrast, in early adolescent group the N-back condition had a significant main effect on the P300 latency at Pz electrode [χ^2^(2) = 15.51, *p* = 0.001, Kendall’s W = 0.32] and no significant effect at the Fz electrode [χ^2^(2) = 2.38, *p* = 0.3, Kendall’s W = 0.05]. The post-hoc analysis indicated that P300 latency at Pz was significantly higher in the two-back condition compared to the zero-back condition (*p* < 0.0001). On the other hand, the P300 latencies are not significantly different between zero-back and one-back conditions (p = 0.154), as well as between one-back and two-back conditions (p = 0.154)

Since the P300 latencies were normally distributed for the Fz electrode in the late adolescents group and the Cz electrode in the early adolescents group, a repeated measures of ANOVA was carried out to investigate the effect of the N-back condition on the P300 latencies. The results showed a significant main effect of N-back conditions at the Fz electrode [*F*(2,46) = 5.66, *p* = 0.006, η^2^ = 0.19] in the late adolescents group and at Cz [*F*(2,34) = 3.37, *p* = 0.046, η^2^ = 0.166] in the early adolescents group. The following post-hoc analysis indicated that the P300 latency at Fz was significantly higher in the one-back compared to zero-back conditions (*p* = 0.03), and the P300 latency at two-back was significantly higher compared to the zero-back condition (*p* < 0.01). But no significant difference was found between one-back and two-back conditions (p = 1). On the other hand, P300 latency at Cz was not significantly different between any of the comparisons, including zero-back and one-back conditions (*p* = 0.08), zero-back and two-back conditions (*p* = 0.07), and one-back and two-back conditions (*p* = 0.840).

In addition, to compare the P300 latencies between groups at each electrode site, the Wilcoxon signed test was carried out separately for three N-back conditions. The results showed no significant difference between groups across N-back conditions at Pz [zero-back (U=313.5, p=0.59), one-back (U=289.5, p=0.97), and two-back (U=316.5, p=0.55)], Cz [zero-back (U=319, p=0.52), one-back (U=274.5, p=0.78), and two-back (U=273.5, p=0.76)], and Fz [zero-back (U=374, p=0.07), one-back (U=249.5, p=0.42), and two-back (U=312, p=0.62)].

### Comparison of P300 amplitude between groups across the electrode sites for maintenance and manipulation conditions

Table 3 shows the mean amplitude and standard deviation for the P300 across electrode sites for maintenance conditions (1 vs. 0), manipulation conditions (2 vs. 1), and maintenance and manipulation conditions (2 vs 0). The P300 amplitude was highest in the maintenance condition and lowest in the manipulation condition in both groups. Further, across electrodes, the P300 amplitude was highest at the electrode Pz, followed by Cz and Fz in both groups. Prior to further statistical analysis, the amplitude data of P300 were subjected to the Shapiro–Wilk test, which indicated the data at the electrode sites Pz, Cz, and Fz were normally distributed in both groups. Thus, a repeated measures of ANOVA was carried out with working memory demand (maintenance, manipulation, and maintenance and manipulation) as the within-subject factor and group as the between-subject factor. The Mauchly’s test of sphericity indicated that the P300 amplitude data violated the sphericity assumptions for all electrode sites, including Pz [χ^2^(2) = 36.146, *p* < 0.001], Cz [χ^2^(2) = 47.37, *p* < 0.001], and Fz [χ^2^(2) = 17.111, *p* < 0.001]. Hence, the following ANOVA main effects are reported with Greenhouse-Geisser corrections.

**Table 3:** Mean P300 amplitude (in µV) and standard deviation (in parenthesis) for late and early adolescent groups across the electrode sites for maintenance, manipulation, combined maintenance and manipulation respectively

Electrode	Maintenance		Manipulation		Combined maintenance and manipulation	
	Late adolescents	Early adolescents	Late adolescents	Early adolescents	Late adolescents	Early adolescents
Pz	0.75 (2.55)	0.39 (2.85)	-1.22 (2.03)	-1.33 (2.60)	-0.46 (2.38)	-0.94 (3.22)
Cz	0.64 (2.11)	0.98 (1.88)	-1.12 (1.61)	-1.20 (1.73)	-0.48 (1.94)	-0.21 (1.71)
Fz	1.77 (4.08)	0.92 (3.61)	1.87 (3.06)	0.42 (3.42)	3.65 (5.51)	1.34 (4.17)

Note: Amplitude in micro volt (µV).

The results indicated that there was no significant interaction between group and working memory processing abilities at all electrode sites, including Pz [*F*(1.28, 59.27) = 0.631, *p* = 0.83, η^2^ = 0.02], Cz [*F*(1.20, 54.23) = 0.203, *p* = 0.70, η^2^ = 0.157], and Fz [*F*(1.519, 69.89) = 0.73, *p* = 0.45, η^2^ = 0.016]. However, the condition has a significant main effect on P300 amplitude at Pz [*F*(1.28, 59.27) = 8.55, *p* < 0.003, η^2^ = 0.157] and Cz electrode [*F*(1.20, 54.23) = 16.22, *p* < 0.001, η^2^ = 0.265]. But, the condition had no significant effect on the P300 amplitude at Fz [*F*(1.519, 69.89) = 2.917, *p* = 0.07, η^2^ = 0.06]. The following post-hoc analysis for the Pz electrode site indicated that the P300 amplitude in the maintenance condition was significantly higher than the manipulation condition (*p* = 0.011) and the combined maintenance and manipulation conditions (*p* = 0.001). However, P300 amplitude between the manipulation condition and the combined maintenance and manipulation conditions did not differ (*p* = 0.44). On the other hand, for the Cz electrode site, the P300 amplitude in the maintenance condition was significantly higher than the manipulation condition (*p* < 0.001) and the combined maintenance and manipulation conditions (*p* < 0.001). Additionally, the P300 amplitude in the combined maintenance and manipulation condition was higher than in the manipulation condition (*p* = 0.024).

## DISCUSSION

Results of the present study showed higher accuracy and shorter reaction time for N-back tasks among late adolescents compared to early adolescents. This finding in the present study is consistent with the results of several studies investigating the effect of age on the N-back task in visual and auditory modality^(49-54)^. The superior performance in late adolescents could be due to enhanced processing speed with age^(55,56)^. The observed effects may be partly explained by age-related changes in white matter microstructure^(57)^. The age-related improvement in accuracy might be attributed to greater neural separation between presented items, as proposed by signal detection theory^(58)^, possibly resulting from a sharpening of neural representations with age. Further, results of the present study showed that performance of adolescents in both groups decreased with increasing N in the N-back task. This finding is also consistent with results of several investigations^(49,59-61)^. Poorer performance in one-back and two-back conditions could be attributed to increased task difficulties^(60)^.

The study found that the P300 amplitude was largest at the electrode site Pz, followed by Cz and Fz in both the groups. Further, the amplitude of P300 was higher in early adolescents compared to late adolescents at Pz across all N-back conditions. However, this difference was not statistically significant. Similar to the findings of present study, many investigations have reported a smaller P300 in older adolescents. In childhood, the amplitude of P300 increases due to maturational changes in the brain, but after the age of 13 years the amplitude decreases till adult values are reached^(35,62-65)^. In contrast, few studies have reported no change in the P300 amplitude among adolescents^(64,66,67)^. In addition to the age-related changes, lower amplitude P300 in late adolescents could be due to difference in skull thickness between the groups. Skull thickness is known to increase slightly with age, especially in the frontal and parietal regions^(52)^. The increase in skull thickness is associated with an opposing effect on P300 amplitudes, the smaller amplitude was found to be associated with thicker skull^(68-69)^. Further, the amplitude of P300 is primarily associated with activities involving attention, memory, and problem-solving^(70)^, and these processing abilities improve significantly from age five to 15 years^(71)^. Information processing ability tends to stabilize around the age of 15 years, showing little change for the amplitude of P300 between late adolescence and adulthood^(38)^. Thus, the reduction in amplitude of P300 in late adolescents in the present study may be associated with the age-related changes in brain, difference in skull thickness, and reduced information processing ability.

The amplitude of P300 was largest in one-back task and smallest in two-back task. One-back and two-back tasks are more difficult to perform compared to zero-back, as cognitive demand increases the reduction in amplitude was expected. The above finding may be due to nature of task involved in zero back, one back and two back conditions. In zero-back condition, a total eight syllables were in random order, and the target syllable was /pa/. Participants had to press response key whenever they heard syllable /pa/. Whereas, in one-back condition participants had to press the response key whenever the previous and current syllables were same. Although the zero-back condition is easier, its repetitive and automatic nature may lead to lower attentional engagement, which may have diminish the strength of the P300 response. Each task has specific demands and captures attention based on these demands^(72)^. This attention then modulates other cognitive systems (e.g., perceptual systems, memory systems, response systems), and according to the multiple resource theory (MRT), every cognitive system has limited resources^(73)^. Based on the type of task, we can infer the resources demanded by the task^(74-77)^. Investigated the effect of different types of auditory stimuli on the P300 component and reported that novel sounds captured more attention and generated a large P300 component compared to simple auditory tones. The above finding is in accordance with^(42,78,79)^, Who attributed lesser P300 amplitudes at 0-back and larger amplitude at 1-back, probably due to differences in attentional demands and updating. In contrast, decrease in P300 amplitude with increasing N back difficulty level was reported^(59,61)^. Additionally, P300 amplitude was greater for targets stimulus than for non-targets, possibly reflecting recognition. Study is in accordance with^(78,80-82)^. The above finding rarely been reported in context with speech stimulus as auditory N back task.

The latency of P300 was slightly shorter in early adolescents compared to late adolescents, however, the difference was not significant. This difference in latency of the P300 between groups could be an effect of maturational changes in the central nervous system. Studies have reported a reduction in the latency of the P300 till 20 years of age. Ute Pfueller et al.^(83)^ showed a reduction in the latency of the P300 between ages 6 and 17, with a slower decline between ages 13 and 17. Other studies have reported that the latency of the P300 reaches its minimum between ages 15 and 20 years^(84-88)^. Further, the present study showed that, the P300 latency was slightly longer in the two-back task compared to one-back and zero-back tasks. This finding is consistent with the results of several investigations^(42,89-91)^. The longer latency of the P300 in two-back condition could be attributed to higher memory load^(42)^ or cognitive load during the two-back task^(91)^. However, not all studies support this trend, Watter et al.^(82)^ did not find a clear relationship between task difficulty and P300 latency^(82)^. Finally, the mean latency and amplitude were not significantly different between groups. This finding could be a due to variability in the maturation of prefrontal cortex among adolescents^(92-95)^. The variability in maturation has been attributed to genetic factors and environmental factors across studies. The environmental factors such as nutrition, sleep habits, exposure to neurotoxins, caffeine, early life stress are known to disrupt normal brain development^(92)^. The variability in maturation could affect cognitive development including working memory and P300^(93)^.

The maintenance process of working memory typically involves storing, monitoring, and matching of incoming stimulus. The amplitude of P300 in maintenance condition (difference of one-back and zero-back) was highest at all the electrode sites compared to maintenance/manipulation condition (two-back and zero-back) and manipulation condition (two-back and one-back). This finding could be attributed to the higher information processing demand and focused resource allocation in the one-back condition compared to zero-back and two-back conditions. In addition, the magnitude of amplitude change could also depend on the attentional effort devoted to processing the stimulus. In contrast Dong Liu, Chunyan Guo, Jin Luo^(96)^ reported that ERPs were significantly more positive during the manipulation phase compared to the maintenance phase at frontal and central regions, but this difference was not observed in parietal regions. There is a scarcity of studies investigating the effect of maintenance and manipulation processes for auditory verbal stimuli using ERP. However, the findings of the present study are consistent with results reported for one-back condition in earlier investigations. The one-back condition is the easy task, hence requires less auditory verbal working memory load and thus yields a better P300 amplitude^(42)^. This aligns with Johnson's^(97)^ observation that any stimulus capturing attention enhances the positive ERP amplitude approximately 300 milliseconds after the stimulus onset.

The manipulation process of working memory involves reordering and updating the information stored in working memory temporarily and it is a complex process. The P300 amplitude was lowest in the manipulation condition compared to maintenance/manipulation and maintenance conditions across electrode sites. This finding may be attributed to the range of diverse characteristics of information processing and wider spread of the resource’s allocation to the conditions in increases auditory verbal working memory load condition. Our present study finding is in accordance with Polich^(9)^, who associated smaller P300 amplitude with reduced performance on a range of diverse characteristics of information processing. Two-back condition being the difficult condition, the auditory verbal working memory load is higher, hence focused attention may be difficult to maintain leading to reduced P300 amplitude^(41)^. Miller et al.^(31)^ and Jaeggi et al.^(32)^ have demonstrated that the two-back condition is more difficult than one-back condition using behavior data and suggested that it requires different processing. An fMRI study revealed that similar brain networks are activated during both maintenance and manipulation conditions, but with differences in the extent and intensity of the activity. Maintenance involved lower and more concentrated activity, whereas manipulation was associated with higher and more dispersed activity^(33)^.

The present study has few limitations. First, results of the present study showed that the amplitude of P300 between groups was not significantly different. The lack of significant group differences may be due to small sample size. Thus, a larger sample could enhance statistical power and the likelihood of detecting significant differences. Second, in literature studies have documented a habituation and dishabituation effects on the amplitude of P300^(98)^. Habituation leads to a reduction in the amplitude of P300, and it typically occurs during the initial stages of recording. However, as the stimulation continues, the rate of amplitude reduction slows and eventually reverse, leading to an increase in amplitude, this process is referred to as dishabituation. The habituation and dishabituation effects were not controlled in the present study. However, a study has reported less habituation effect in adolescents compared to children^(83)^. Third, gender distribution was unequal due to the availability of participants. However, prior studies have consistently reported no gender differences for the latency and amplitude of P300^(93)^. Due to the small sample size of current investigation gender-based comparison was not carried out. Fourth, several classification systems are available to classify adolescents into different groups. The current study uses age groupings derived from *Development Through Life: A Psychosocial Approach* by Newman and Newman, which outlines human development across the lifespan—from infancy to old age. This framework is heavily influenced by Erik Erikson’s psychosocial theory and integrates it with modern research and practical insights^(44)^. However, given that P300 variations are closely linked to neurocognitive development. In future it would be more appropriate to use age ranges that reflect key stages of brain maturation—such as 12–14 years (early adolescence), 15–17 years (mid-adolescence), and 18–24 years (late adolescence to early adulthood). Adopting this classification would enhance the precision of age-related analyses of evoked potential.

### Clinical implications and practical applicability

By comparing P300 amplitudes and latencies in maintenance (simple recall) vs. manipulation (mental reordering) conditions, researchers can assess the developmental changes of working memory functions between early (e.g., 12–18 years) and late adolescents (e.g., 12–24 years). Differences in the P300 responses may indicate greater neural efficiency or cognitive resource allocation in older adolescents, reflecting maturation of the prefrontal cortex. Understanding age-specific neural responses helps design age-appropriate memory training or executive function programs in educational or clinical settings. Atypical P300 patterns may signal developmental delays or neurocognitive disorders (e.g., ADHD, learning disabilities), prompting early intervention. Since the task is auditory, the results are especially relevant for evaluating listening-related cognitive load, which is critical in classroom environments where students must process verbal information under distraction. Comparing maintenance and manipulation conditions helps isolate how adolescents handle increasing cognitive demands, aiding in optimizing learning environments. The findings contribute to normative data on adolescent brain responses, useful in clinical neuropsychological assessments. Given that P300 is sensitive to attention and executive control, this paradigm may be used to monitor adolescents at risk of conditions such as anxiety, depression, or substance use, where cognitive control is affected.

## CONCLUSION

The present study shows the P300 amplitude is not significantly different between early and late adolescents across n-back tasks used in the present investigation. Further, the P300 amplitude was largest in maintenance conditions and lowest in manipulation conditions.
